# Promising antimalarial hits from phenotypic screens: a review of recently-described multi-stage actives and their modes of action

**DOI:** 10.3389/fcimb.2023.1308193

**Published:** 2023-12-15

**Authors:** Annie-Peiyuan Luo, Carlo Giannangelo, Ghizal Siddiqui, Darren J. Creek

**Affiliations:** Drug Delivery Disposition and Dynamics, Monash Institute of Pharmaceutical Sciences, Monash University, Parkville, VIC, Australia

**Keywords:** *Plasmodium falciparum*, antimalarial, phenotypic screening, mode of action, target identification

## Abstract

Over the last two decades, global malaria cases caused by *Plasmodium falciparum* have declined due to the implementation of effective treatments and the use of insecticides. However, the COVID-19 pandemic caused major disruption in the timely delivery of medical goods and diverted public health resources, impairing malaria control. The emergence of resistance to all existing frontline antimalarials underpins an urgent need for new antimalarials with novel mechanisms of action. Furthermore, the need to reduce malaria transmission and/or prevent malaria infection has shifted the focus of antimalarial research towards the discovery of compounds that act beyond the symptomatic blood stage and also impact other parasite life cycle stages. Phenotypic screening has been responsible for the majority of new antimalarial lead compounds discovered over the past 10 years. This review describes recently reported novel antimalarial hits that target multiple parasite stages and were discovered by phenotypic screening during the COVID-19 pandemic. Their modes of action and targets in blood stage parasites are also discussed.

## Introduction

Malaria, a bloodborne protozoan infection, is a continuous threat to global health. Among the six *Plasmodium* species that can infect humans, *Plasmodium falciparum* causes the most severe symptoms (Burrows et al., 2013; Ashley et al., 2018). From 2000 to 2017, the global *P. falciparum* incidence declined from 232.3 million to 193.9 million, and deaths declined by 42.5% (Weiss et al., 2019). This was achieved due to the collective impacts of insecticide-treated nets (ITNs), insecticide sprays and artemisinin-based combination therapy (ACT) (Bennett et al., 2017; Weiss et al., 2019). However, new challenges have arisen. Resistance to artemisinin has emerged in Southeast Asia, South America, Sub-Saharan Africa and Papua New Guinea (Chenet et al., 2016; Mathenge et al., 2020; Miotto et al., 2020; Uwimana et al., 2020). Although this has not directly resulted in widespread treatment failures for the recommended ACTs, the impact of artemisinin resistance is exacerbated by resistance to the ACT partner drugs. Meanwhile, the COVID-19 pandemic has disrupted the distribution of ITNs and the administration of seasonal malaria chemoprophylaxis as limited healthcare resources were shifted towards the control of COVID-19 (Hussein et al., 2020; Heuschen et al., 2021). Furthermore, overlapping symptoms between COVID-19 and malaria has made accurate diagnosis more challenging (Zawawi et al., 2020). This mis-diagnosis has also been shown to increase sales and misuse of non-prescription antimalarials, potentially exacerbating the emergence of antimalarial resistance (Makanjuola et al., 2021). Since resistance to all frontline antimalarials has rapidly emerged and the COVID-19 pandemic has caused an unexpected disruption in progress toward malaria elimination, there is an even greater need to develop novel antimalarials.

**Figure 1:**
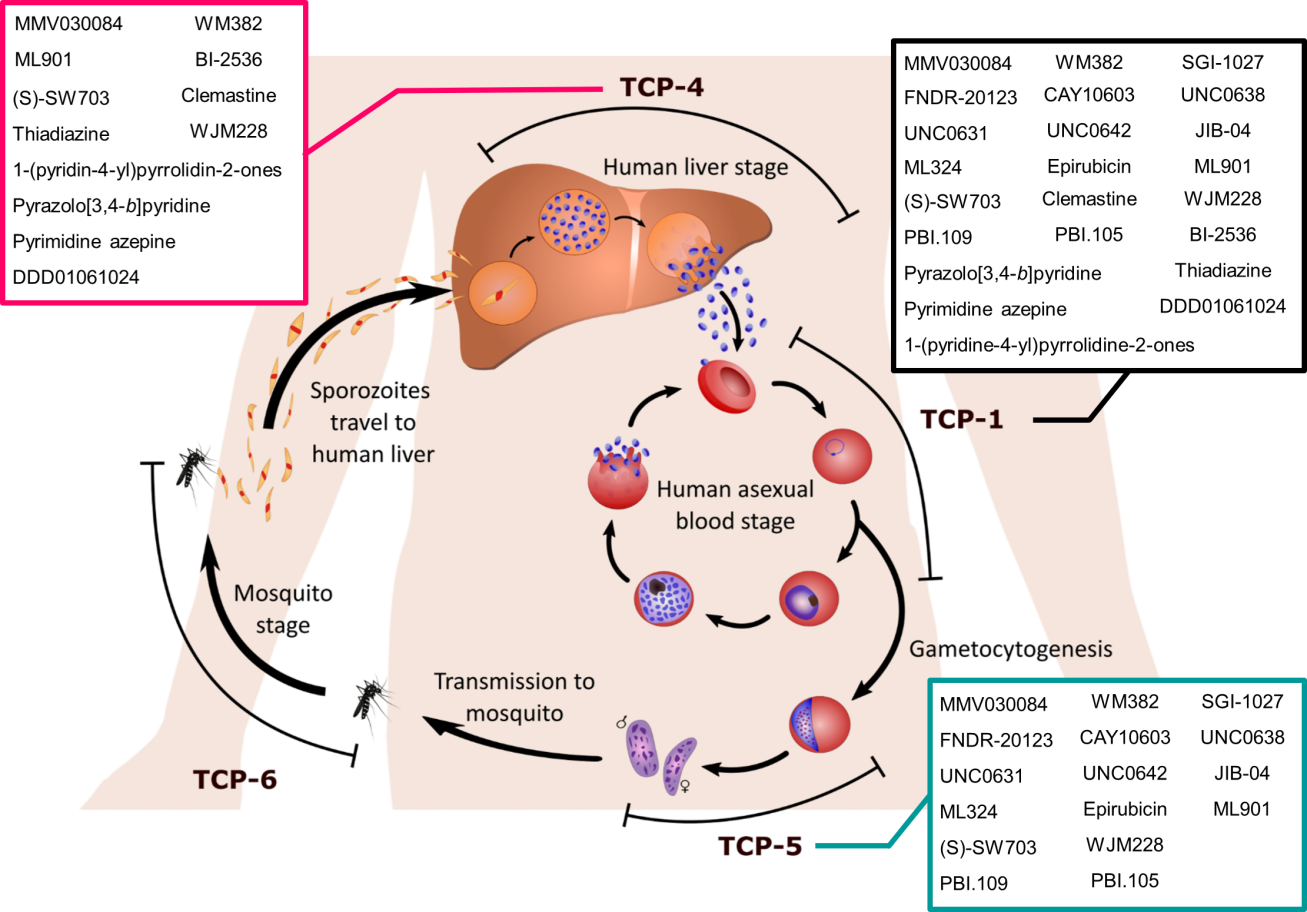
The life cycle stages of *Plasmodium* parasites. Sporozoites are introduced into the human body via a blood meal of a female *Anopheles* mosquito. Sporozoites invade the human liver and replicate asexually for 1-2 weeks whilst the host remains asymptomatic. Compounds targeting liver stage *Plasmodium* parasites satisfy the target candidate profile 4 (TCP-4) criteria. Once the parasitaemia reaches a threshold, the liver schizonts burst into hundreds of merozoites that invade host red blood cells (RBCs). At this stage of the *Plasmodium* lifecycle, the host will become symptomatic and require effective antimalarial treatment to clear the asexual blood stage parasitaemia (TCP-1). The asexual blood stage consists of rings, trophozoites, schizonts and merozoites. Whilst *Plasmodium* parasites amplify their number in the asexual replication stage, a portion of rings commit to sexual differentiation into gametocytes via a process called gametocytogenesis. Compounds with TCP-5 display transmission-blocking activity against gametocyte parasites, the sexual stage. Once these gametocytes are ingested by mosquitoes, the male and female gametes fuse into a zygote, which further develops into an ookinete. The ookinete migrates across the mosquito gut epithelium to form an oocyst, which releases sporozoites after a series of replication events. Transmission-blocking activity against the mosquito vector is described as TCP-6. Sporozoites eventually travel to the mosquito salivary gland and prepare for the next infection, thus completing the lifecycle. Compounds satisfying TCP-1, TCP-4 and TCP-5 are listed in the black, magenta and turquoise boxes respectively.

Six target candidate profiles (TCPs) have been proposed to assist in the evaluation of potential antimalarial candidates (Burrows et al., 2017). In relevance to *P. falciparum* infections, three TCPs describe antimalarial activity against the three distinct stages of the parasite life cycle (**Figure 1**), the symptomatic asexual blood stage (TCP-1), asymptomatic liver stage (TCP-4) and sexual stage (TCP-5). Ideally, a single-dose treatment regimen would be the most beneficial malaria treatment, however, this is difficult to achieve with one antimalarial. Hence, it is recommended to combine two or more antimalarials, each of which satisfies multiple TCPs or in other words, demonstrates multi-stage activities. Considerable advances in high throughput screening (HTS) technologies have expanded the detection range for antimalarial activities from asexual parasites to other stages of their life cycle (Hovlid and Winzeler, 2016). Here, we review a panel of multi-stage active (at least two TCPs) antimalarial hit compounds discovered from HTS with identified modes of action (MOAs), which were published during the COVID-19 pandemic (March 2020 – May 2023) (**Table 1**).

**Table 1:** Summary of novel multi-stage active compounds discovered in HTS during the COVID-19 pandemic.

Compound	Chemical Structure	Multi-stage activity IC_50_ (µM)			Mode of action^IV^	Reference
		TCP1^I^	TCP4^II^	TCP5^III^		
MMV030084	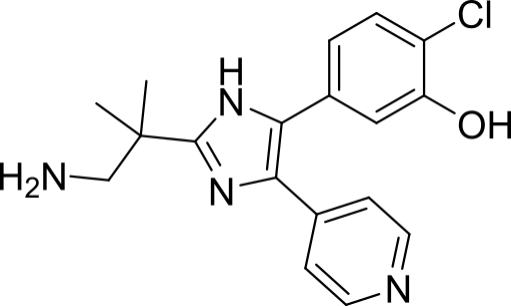	0.12	0.199	0.141	Inhibits *Pf*PKG in the egress cascade and subsequently inhibits merozoite egress	(Vanaerschot et al., 2020)
WM382	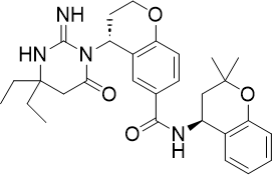	0.0006	100 mg/kg^a^	0.0025	Inhibits PMIX and PMX in the egress cascade, disrupting merozoite egress and invasion	(Favuzza et al., 2020)
SGI-1027	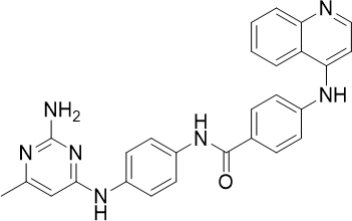	0.045	N/A	0.014	Inhibits *Pf*DNMT, which disrupts the functioning of promotors and exons for virulence genes	(Vanheer et al., 2020)
FNDR-20123	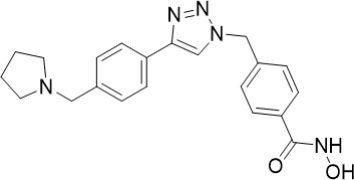	0.041	N/A	0.19	Inhibits *Pf*HDAC and disturbs the histone acetylation equilibrium, which further disrupts the parasitic	(Potluri et al., 2020)
CAY10603	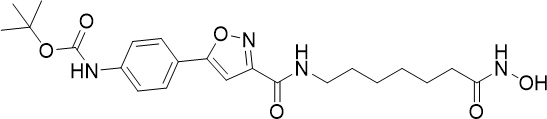	0.37	N/A	1.3		(Coetzee et al., 2020)
UNC0638	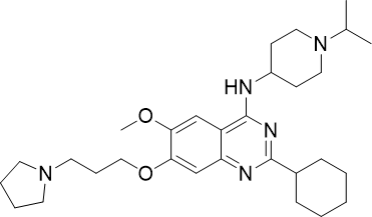	0.0283	N/A	0.442	Inhibits *Pf*HKMT and disturbs the histone methylation equilibrium, which further disrupts the parasitic gene expression	(Coetzee et al., 2020)
UNC0631	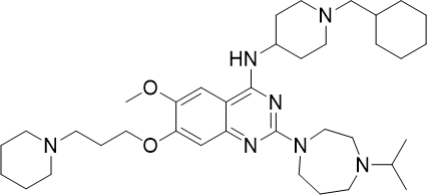	0.024	N/A	0.014		(Vanheer et al., 2020)
UNC0642	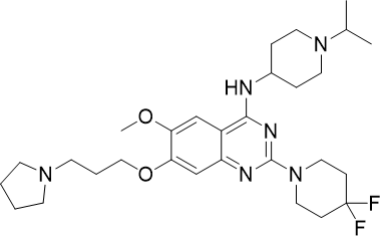	0.016	N/A	0.014		(Vanheer et al., 2020)
JIB-04	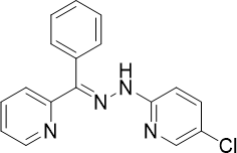	0.535	N/A	0.14	Inhibits *Pf*HDM and disturbs the histone methylation equilibrium, which further disrupts the parasitic gene expression	(Vanheer et al., 2020)
ML324	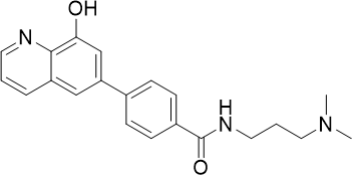	2.1	>5	0.077		(Reader et al., 2021)
Epirubicin	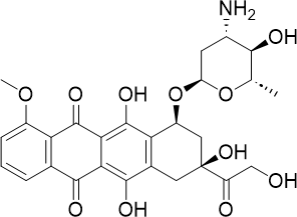	0.111	N/A	0.39	Inhibits *Pf*GryA, which further disrupts DNA replication, transcription and repair	(Ferreira Letícia et al., 2020)
ML901	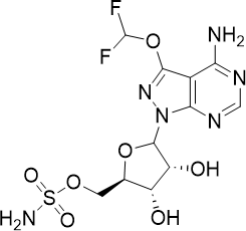	0.002	0.013*^Pf^*	0.13	Inhibits *PfYRS* and hence parasite protein translation	(Xie et al., 2022)
1-(pyridin-4-yl)pyrrolidin-2-ones	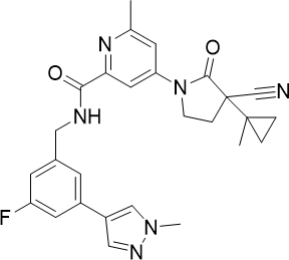	0.01	0.013*^Pf^*	Inactive	Inhibits *Pf*cPRS and downstream protein synthesis	(Okaniwa et al., 2021)
BI-2536	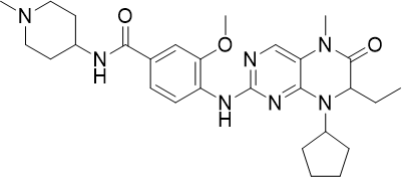	0.178	0.367	N/A	Inhibits *Pf*NEK3 and potentially a *Pf*MRS	(Bohmer et al., 2023)
(S)-SW703	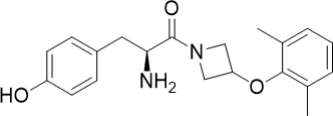	0.78	0.57	1	Resistance to (S)-SW703 is associated with *Pf*CARL and *Pf*AT1	(Imlay et al., 2023)
Clemastine	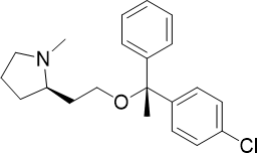	2.5	6.3	N/A	Disrupts microtubule biogenesis and induces abnormal cytoskeletal morphology	(Lu et al., 2020)
Pyrazolo[3,4-*b*]pyridine	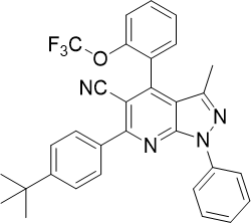	0.278	0.57	>25	Inhibits cytochrome *bc_1_* complex, disrupting the downstream *de novo* pyrimidine synthesis pathway	(Eagon et al., 2020)
Thiadiazine	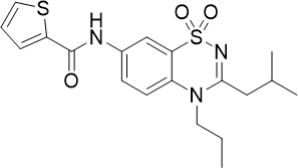	0.219	0.003	N/A		(Dorjsuren et al., 2021)
Pyrimidine azepine	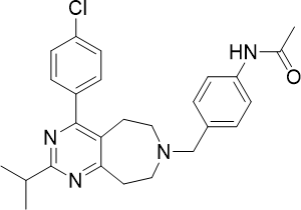	1.5	0.71	N/A	Inhibits cytochrome *bc_1_* complex and DHODH, disrupting the downstream *de novo* pyrimidine synthesis pathway	(Dorjsuren et al., 2021)
DDD01061024	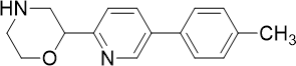	0.344	0.375	N/A	Inhibits cytochrome *bc_1_* complex	(Abraham et al., 2020)
WJM228	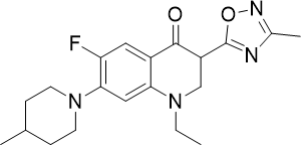	0.02	0.001	0.56	Inhibits cytochrome *bc_1_* complex and potentially DHODH	(Nguyen et al., 2023)
PBI.109	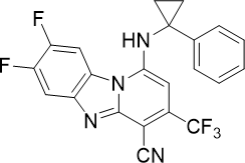	0.0537	N/A	0.102	Inhibits haemozoin formation	(Leshabane et al., 2021)
PBI.105	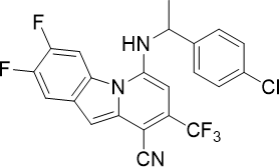	0.0942	N/A	0.569		(Leshabane et al., 2021)

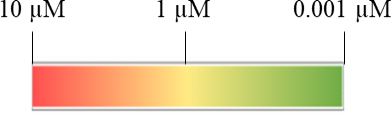

^I^ Values represent the *in vitro* potency of compounds against asexual blood stage *P. falciparum.*

^II^ Values represent the potency of compounds against *in vitro* liver cells infected by *P. berghei* or ^Pf^ *P. falciparum* or ^a^ chemoprotection effect in *P. berghei*-infected mice in vivo (the ED_50_ value is not available).

^III^ Values represent the potency of compounds against gametocyte growth, gamete development, oocyst formation and ookinete development of *P. falciparum*.

^IV^ Mode of action against the asexual blood stage *P. falciparum*.

Antimalarial activity against different life cycle stages is annotated with a colour gradient where green represents low nanomolar potency and yellow-to-red represents micromolar potency. The grey colour is assigned to compounds that are inactive against a life cycle stage, or their activity against that life cycle stage has not been assessed.

## Inhibiting merozoite egress and invasion

Several phenotypically active compounds identified over the past three years have been found to target the parasite processes of egress and invasion. Once asexual blood stage parasites reach the mature schizont stage, merozoites are released from infected red blood cells (RBCs) via a process called egress (Tan and Blackman, 2021). Following egress, the free merozoites invade new RBCs to continue the asexual life cycle. Since merozoite egress and invasion are essential for the continuation of the *P. falciparum* life cycle, the egress and invasion machinery are promising antimalarial targets.

The trisubstituted imidazole, MMV030084, was a multi-stage active compound that was identified in a phenotypic screen by Vanaerschot et al. (Vanaerschot et al., 2020). It displayed nanomolar potency against asexual (TCP-1) and sexual blood stages (TCP-5) *P. falciparum*, as well as liver stage (TCP-4) *P. berghei* (**Figure 1**) (Vanaerschot et al., 2020). MMV030084 was found to target *P. falciparum* cGMP-dependent protein kinase (*Pf*PKG) in asexual stages by interacting with the enzyme’s ATP binding site (Vanaerschot et al., 2020). *Pf*PKG initiates the parasite egress cascade in response to an increased level of intracellular cGMP (Taylor et al., 2010; Collins et al., 2013). Consistent with *Pf*PKG being the target, MMV030084 treatment resulted in abnormal schizont morphology and it halted the progression of parasites into rings (Vanaerschot et al., 2020). Additionally, the transmission-blocking activity of MMV030084 was likely due to inhibition of *Pf*PKG during the sexual blood stage, which prevented the activation and emergence of gametocytes (Diaz et al., 2006; McRobert et al., 2008; Taylor et al., 2010; Brochet and Billker, 2016). The liver-stage activity of MMV030084 against *P. berghei* potentially resulted from inhibiting *Pb*PKG and subsequently delaying the development and egress of hepatic merozoites. However, the direct binding of MMV030084 *Pb*PKG needs further validation and the translation of this liver-stage activity against *P. falciparum* requires further investigation.

WM382 is a dual inhibitor of plasmepsin IX (PMIX) and plasmepsin X (PMX) that prevents both merozoite egress and invasion (Favuzza et al., 2020). It was optimized based on two hits from an aspartic protease inhibitor library and demonstrated nanomolar activity against asexual blood stage (TCP-1) parasites with no cross-resistance to existing frontline antimalarials (Favuzza et al., 2020). WM382 also reduced the viability and infectivity of liver stage merozoites (TCP-4) and inhibited oocyst development (TCP-5) (**Figure 1**) (Favuzza et al., 2020). Hence, WM382 has both suppressive prophylactic and transmission-blocking potential, in addition to its ability to cure an active malaria infection. PMIX and PMX were identified as the targets for WM382 by cellular thermal shift assay and validated by improved potency observed against PMIX and PMX knockdown parasites (Favuzza et al., 2020). PMX catalyses the activation of proteins and enzymes involved in the egress cascade and merozoite invasion, whereas PMIX is only essential for merozoite invasion (Yeoh et al., 2007; Nasamu et al., 2017; Pino et al., 2017; Thomas et al., 2018; Favuzza et al., 2020). In addition to preventing the asexual blood stage reproduction of *P. falciparum* parasites, dual inhibition of PMIX and PMX has also been shown to delay hepatic merozoite egress and prevent development of fertile gametes (Pino et al., 2017).

## Disrupting epigenetic regulation

In *P. falciparum*, epigenetic regulation controls the expression of genes for essential parasite processes, including metabolic activity, immune evasion, merozoite invasion, gametocytogenesis and sexual differentiation (Abel and Le Roch, 2019; Hammam et al., 2021). Hence, compounds that disrupt epigenetic regulation are detrimental to the survival of asexual and sexual parasites.

SGI-1027, a DNA methyltransferase inhibitor (DNMT), showed low-nanomolar potency against asexual blood stage (TCP-1) parasites and early-stage gametocytes (TCP-5) (**Figure 1**) (Vanheer et al., 2020). Similarly, other DNMT inhibitors have been reported to demonstrate low-nanomolar potency against asexual blood stage *P. falciparum* (Nardella et al., 2020). *P. falciparum* DNMT (*Pf*DNMT) is an epigenetic regulator that methylates parasite DNA and controls gene expression (Lucky et al., 2023). Inhibition of *Pf*DNMT may disrupt parasite metabolism and subsequently impact stress response and merozoite invasion (Hammam et al., 2021; Lucky et al., 2023). However, further validation is required to demonstrate direct inhibition of *P. falciparum* DNMT (*Pf*DNMT) by SGI-1027.

Histone acetylation and methylation provide essential epigenetic regulation mechanisms for parasite growth and development throughout asexual blood stages (TCP-1) and gametocytogenesis (TCP-5) (**Figure 1**) (Chaal et al., 2010; Gupta and Bozdech, 2017; Neveu et al., 2020). The acetylation state of histone 3 lysine 9 (*Pf*H3K9) and histone 3 lysine 14 (*Pf*H3K14) is governed by histone acetyltransferases and histone deacetylases (*Pf*HDAC), which regulate the expression of *P. falciparum* genes for essential parasite pathways such as merozoite invasion and sexual differentiation (Abel and Le Roch, 2019). FNDR-20123 was identified in a screen of a HDAC inhibitor library by Potluri et al., and was found to inhibit multiple *Pf*HDAC isoforms at nanomolar concentration. FNDR-20123 showed low-nanomolar activity against asexual blood stage (TCP-1) parasites and male gametocytes (TCP-5) (Potluri et al., 2020). A 4-day treatment of 50 mg/kg FNDR-20123 reduced parasitaemia by 50-fold in infected mice. However, FNDR-20123 was 10-fold more selective towards human HDAC than *Pf*HDAC, indicating that further chemical optimisation is needed to limit the risk of host cell toxicity.

CAY10603 and UNC0638 were identified as hits from the Cayman Epigenetics library with nanomolar activity against asexual blood stage (TCP-1) of *P. falciparum* and no cross-resistance with chloroquine, pyrimethamine or cycloguanil (Coetzee et al., 2020). CAY10603 was equally active against early and late-stage gametocytes (TCP-5), whereas UNC0638 was more active against late-stage gametocytes (**Figure 1**). Although further affinity- or functional-based validation is required, CAY10603 and UNC0638 are predicted to inhibit *Pf*HDAC and *P. falciparum* histone methyltransferase (*Pf*HMT) respectively. *Pf*HMT and histone demethylase (*Pf*HDM) control the methylation state of *Pf*H3K9 and histone 3 lysine 4 (*Pf*H3K4), regulating gene expression that is essential for asexual and sexual blood stage parasites (Malmquist et al., 2015). Hence, two *Pf*HMT inhibitors, UNC0631 and UNC0642, also showed nanomolar potency against asexual (TCP-1) *P. falciparum* and early-stage gametocytes (TCP-5) (**Figure 1**) (Vanheer et al., 2020).

In a phenotypic screen performed by Vanheer et al, JIB-04 showed higher potency against early-stage gametocytes (TCP-5) than asexual blood stage (TCP-1) parasites (**Figure 1**) (Vanheer et al., 2020). JIB-04 competitively inhibits the Jumonji-domain-containing *Pf*HDM, disrupting the histone methylation equilibrium, leading to gene dysregulation and eventually parasite death (Matthews et al., 2020; Vanheer et al., 2020). Interestingly, ML324 (MMV1580488) shared the same proposed target as JIB-04 but displayed a different antimalarial profile (Reader et al., 2021). ML324 was potent against late-stage gametocytes (TCP-5) whilst showing minimal activity against early-stage gametocytes and asexual blood stage parasites (**Figure 1**). Exposure to ML324 resulted in an increased level of methylated *Pf*H3K9 and the associated changes in gene expression resembled JIB-04 treatment (Matthews et al., 2020; Reader et al., 2021).

Epirubicin is predicted to be an antimalarial hit via computational chemogenomics (Ferreira Letícia et al., 2020). It demonstrated nanomolar potency against asexual blood stage (TCP-1) *P. falciparum* and blocked the *in vitro* conversion from sexual stage *P. berghei* to ookinetes (IC_50_ 0.39 µM). Epirubicin also reduced 53% of *P. vivax* oocyst formation (TCP-5) in infected mosquitoes’ midguts in membrane feeding assays (**Figure 1**). *P. falciparum* Gyr subunit A (*Pf*GyrA) was predicted to be the target of epirubicin by molecular modelling and docking (Ferreira Letícia et al., 2020). *Pf*Gyr is a type II topoisomerase which releases tension in the supercoiled double stranded DNA and hence facilitates DNA replication (Nagano et al., 2014; Tan et al., 2021). *Pf*GyrA is also essential for the structural integrity of the apicoplast (Goodman and McFadden, 2013; Nagano et al., 2014; Milton and Nelson, 2016; Tan et al., 2021). Resistance selection for epirubicin in *Saccharomyces cerevisiae* resulted in downregulation of *alg7*, which encodes for the dolichol phosphate N-acetylglucosamine-1-phosphotransferase (Ferreira Letícia et al., 2020). Since *P. falciparum* has a homologous *alg7* gene that is thought to be essential, epirubicin is speculated to interfere with protein glycosylation in *P. falciparum* (Zhang et al., 2018; Böhme et al., 2019; Chappell et al., 2020). However, due to potential toxicity to mammalian cells, epirubicin requires further chemical optimisation to be a clinically useful antimalarial.

## Inhibiting protein synthesis

In protein translation, amino acids are shuttled to the ribosomes by their allied transfer RNAs (tRNAs) and assembled into polypeptide chains based on alignment of the tRNA anticodons with the codons on the messenger RNA. Aminoacyl tRNA synthetases (aaRSs) are key upstream enzymes of protein translation which catalyse the formation of amino acid-adenosine 5’ monophosphate (AMP) intermediates and the conjugation of these amino acids to their corresponding tRNAs with the release of AMP (Xie et al., 2023). Key differences in conserved motifs of *P. falciparum* aaRSs (*Pf*aaRSs) and human aaRSs were reported, suggesting *Pf*aaRSs to be promising antimalarial targets during the liver, asexual and sexual blood stages of the parasites (Bhatt et al., 2009; Xie et al., 2023).

ML901, a pyrazolopyrimidine sulfamate, was identified from a screen of the Takeda compound library with nanomolar potency against asexual blood stage (TCP-1), liver stage (TCP-4) and sexual stage (TCP-5) *P. falciparum* (**Figure 1**) (Xie et al., 2022). In addition to its multi-stage activity, ML901 is 800- to 5000-fold less toxic to human cells. ML901 structurally resembles AMP and it inhibits *P. falciparum* tyrosine tRNA synthetase (*Pf*YRS) by hijacking *Pf*YRS-bound tyrosine after AMP is released. LC-MS based methods detected ML901-tyrosine conjugates produced by *Pf*YRS but not by its human homolog, suggesting that ML901 selectively inhibits *Pf*YRS over human YRS (Xie et al., 2022).

The cytoplasmic prolyl-tRNA synthetase (*Pf*cPRS) is essential for *P. falciparum* development in the asexual blood stage (TCP-1) and liver stage (TCP-4) (**Figure 1**) (Herman et al., 2015). In a screen for *Pf*cPRS competitive inhibitors at the ATP-binding site, 1-(pyridine-4-yl)pyrrolidine-2-ones showed nanomolar potency against asexual blood stage and liver stage *P. falciparum* (Okaniwa et al., 2021). Similar to how ML901 prevents the conjugation of tyrosine with its cognate tRNA, 1-(pyridine-4-yl)pyrrolidine-2-ones inhibit the proline-tRNA formation and hence disrupt downstream protein synthesis. However, 1-(pyridine-4-yl)pyrrolidine-2-ones showed off-target inhibition of the human adenosine A3 receptor (IC_50_ 1.8 µM), suggesting that further optimisation is required to reduce potential host toxicity (Okaniwa et al., 2021).

The HMS LINCS library was screened for kinase inhibitors with antimalarial activity and BI-2536 demonstrated low nanomolar potency against asexual blood stage (TCP-1) and liver stage (TCP-4) *P. falciparum* (**Figure 1**) (Bohmer et al., 2023). BI-2536 inhibits Polo-like kinase 1, an important cell division regulator in humans, however, as no *Plasmodium* homologs have been identified, an alternative target is likely responsible for its antimalarial activity (Archambault and Glover, 2009; Lian et al., 2018; Bohmer et al., 2023). BI-2536 was shown to inhibit *P. falciparum* NIMA related kinase 3 (*Pf*NEK3), however, *Pf*NEK3 is not essential for asexual blood parasites but rather highly expressed in gametocytes (Lye et al., 2006; Zhang et al., 2018). *In vitro* selection for BI-2536 resistance identified mutations in methionyl-tRNA synthetase (*Pf*MRS) and other enzymes involved in the tRNA aminoacylation process, suggesting that BI-2536 potentially inhibits protein translation (Bohmer et al., 2023). However, further investigation is required to validate the direct inhibition of *Pf*MRS by BI-2536.

## Disrupting protein trafficking and cellular scaffolding

Similar to other eukaryotic cells, the endoplasmic reticulum (ER) and Golgi apparatus of *P. falciparum* process and package proteins before they are transported to their destination. Some of these proteins are exported outside of the parasite and modify the host RBC membrane, which assists merozoite egress and allows the parasite to evade the host immune system. Some other proteins exported to the host RBC cytoplasm may direct exported protein trafficking, remodel lipids and detoxify haem, in order to make the RBCs more habitable for parasite growth (Cooke et al., 2004). Hence, compounds that disrupt parasitic protein processing in the ER, Golgi apparatus and host RBC can significantly inhibit parasite growth and development.

A tyrosine amide, (S)-SW703 showed a fast-killing profile against asexual blood stage (TCP-1) *P. falciparum* in a HTS (Imlay et al., 2023). It also showed liver-stage (TCP-4) activity and transmission-blocking (TCP-5) activity (**Figure 1**). Resistance selection with (S)-SW703 led to point mutations in the *P. falciparum* cyclic amine resistance transporter (*Pf*CARL) and the acetyl-CoA transporter 1 (*Pf*AT1). The function of *Pf*CARL remains unknown, however, it is not essential for parasite growth (LaMonte et al., 2016). Although the exact function of *Pf*AT1 remains elusive, it is generally accepted to transport acetyl-CoA across the ER/Golgi membrane for downstream protein packaging and trafficking (Martin et al., 2005). Parasites with mutations in *Pf*AT1 showed impaired fitness and grew at a slower rate compared to wildtype parasites, suggesting *Pf*AT1 is important but not essential (Lim et al., 2016). *Pf*CARL and *Pf*AT1 were shown to mediate the resistance mechanisms to multiple compounds with unrelated chemical structures (LaMonte et al., 2016; Lim et al., 2016; Magistrado et al., 2016). Hence, mutations in *Pf*CARL and *Pf*AT1 are more likely to be associated with the resistance mechanism rather than being the direct targets of (S)-SW703. Further target identification studies are required to elucidate the binding target of (S)-SW703.

A few FDA-approved antihistamines have exhibited antimalarial activity against blood stage *P. falciparum* (Chong et al., 2006; Derbyshire et al., 2012; Milner et al., 2012). Since the *Plasmodium* genome does not encode for a homologous protein to mammalian histamine-1 receptor, these antihistamines are hypothesized to act on other *Plasmodium-*specific targets (Derbyshire et al., 2012). For example, clemastine showed single-digit micromolar potency against liver stage (TCP-4) and asexual blood stage (TCP-1) parasites (**Figure 1**) (Lu et al., 2020). Proteomics-based target deconvolution of clemastine revealed the *P. falciparum* chaperonin containing T-complex protein 1 delta subunit (*Pf*CCT4) as the likely target, indicated by stabilization of *Pf*CCT4 when challenged by heating and exposure to denaturant (Lu et al., 2020). *Pf*CCT4 has been shown to be essential for asexual parasite development, particularly in rings and trophozoites where the maximal activity of clemastine was observed (Spillman et al., 2017; Lu et al., 2020). There has been a debate about whether *P. falciparum* CCT4 mediates the trafficking of parasite proteins in the host RBC or if it solely functions within the parasite cytosol (Mbengue et al., 2013; Mbengue et al., 2015; Spillman et al., 2017). Nevertheless, reduced microtubule biogenesis and abnormal cytoskeletal morphology were observed in parasites treated with clemastine. Similar observations were reported when the mammalian CCTs were inhibited (Willison, 2018; Grantham, 2020; Lu et al., 2020). Overall, clemastine combines multi-stage activity with a novel MOA and an extensively studied safety profile in humans.

## Inhibiting the mitochondrial electron transport chain

In blood stage *P. falciparum*, the mitochondrial electron transport chain (mtETC) is crucial for maintaining the precursor pool for *de novo* pyrimidine synthesis (Barton et al., 2010; Rodrigues et al., 2010; Nixon et al., 2013b). Inhibition of the mtETC cytochrome *bc_1_* complex disrupts pyrimidine synthesis and hence suppresses parasite growth during the asexual blood stage and liver stage (Nixon et al., 2013a; David Hong et al., 2018; Okada-Junior et al., 2018; Song et al., 2018; Wojnarski et al., 2019).

Pyrazolo[3,4-*b*]pyridine is a cytochrome *bc_1_* inhibitor which binds to the quinol oxidation (Q_o_) binding site (Eagon et al., 2020). It showed nanomolar potency in a HTS and no cross resistance with chloroquine, pyrimethamine, cycloguanil or quinine in asexual blood stage (TCP-1) parasites (Eagon et al., 2020). Pyrazolo[3,4-*b*]pyridines also showed low-micromolar potency against liver stage (TCP-4) parasites (**Figure 1**). Whilst both pyrazolo[3,4-*b*]pyridine and atovaquone bind to the Q_o_ site of the cytochrome *bc*_1_ complex, *in silico* molecular docking predicted pyrazolo[3,4-*b*]pyridine to fit in a different orientation to atovaquone (Siregar et al., 2015; Eagon et al., 2020). It is worth investigating whether this different binding mechanism of pyrazolo[3,4-*b*]pyridine would conserve its antimalarial activity against atovaquone-resistant parasites.

Pyrimidine azepine and thiadiazine demonstrated activity against both asexual blood stage (TCP-1) and liver stage (TCP-4) *P. falciparum* (**Figure 1**) (Dorjsuren et al., 2021). *In vitro* resistance selection for pyrimidine azepine and thiadiazine resulted in four mutations within or near the Q_o_ site of the *P. falciparum* cytochrome *bc_1_* complex, whilst being distant from the quinol reduction (Q_i_) site (Barton et al., 2010; Dorjsuren et al., 2021). Furthermore, the pyrimidine azepine-resistant parasite line and one thiadiazine-resistant parasite line showed cross-resistance to atovaquone, confirming *P. falciparum* cytochrome *bc_1_* complex as its target. Interestingly, the other two thiadiazine-resistant parasite lines became more susceptible to atovaquone, suggesting that these thiadiazines may have a different binding mechanism to atovaquone despite sharing the common Q_o_ binding site. Furthermore, these two thiadiazine-resistant lines were hypersensitive to DSM265, an inhibitor of dihydroorotate dehydrogenase (DHODH) (Phillips et al., 2015). DHODH is an essential enzyme in the pyrimidine biosynthesis pathway, the function of which relies on the production of oxidized coenzyme Q by the cytochrome *bc_1_* complex. These findings further confirm parasite cytochrome *bc_1_* as the likely antimalarial target of thiadiazine antimalarials.

DDD01061024 is a potential *P. falciparum* cytochrome *bc_1_* inhibitor discovered from the Global Health Chemical Diversity Library (Abraham et al., 2020). It showed nanomolar potency against asexual blood stage (TCP-1) and liver stage (TCP-4) *Plasmodium* parasites (**Figure 1**). Resistance selection for DDD01061024 resulted in a point mutation at the same position as one of the thiadiazine-resistant strains (Dorjsuren et al., 2021). Similarly, DDD01061024-resistant parasites were less susceptible to atovaquone, suggesting *P. falciparum* cytochrome *bc_1_* to be its target.

WJM228, a 7-N-substituted-3-oxadiazole quinolone, was screened out of the Janssen Jumpstarter library with nanomolar potency against asexual (TCP-1), sexual blood stage (TCP-5) and liver stage (TCP-4) *P. falciparum* (**Figure 1**) (Phillips et al., 2015). Resistance selection for WJM228 led to the V259L point mutation on the cytochrome *bc*_1_ Q_o_ binding site, the same mutation which resulted in atovaquone and DDD01061024 resistance (Nixon et al., 2013a; Phillips et al., 2015; Dorjsuren et al., 2021). Furthermore, WJM228 showed reduced susceptibility against DHODH inhibitor-resistant *P. falciparum* (Phillips et al., 2015). These findings suggest that WJM228 also inhibits parasite growth by interfering with the mtETC.

## Inhibiting the haem detoxification process

Blood stage *Plasmodium* parasites contain a unique organelle known as the food vacuole, which is the site of digestion of host cell haemoglobin (Bannister et al., 2000). Haemoglobin digestion is an essential metabolic process and a key point of vulnerability during asexual blood stage development. The parasite digests up to 75% of host cell haemoglobin to provide amino acids for parasitic protein synthesis (Ecker et al., 2012). However, this process releases free haem that is toxic to the parasite. Hence, *Plasmodium* parasites have evolved a unique haem detoxification mechanism in which free haem is polymerised into insoluble hemozoin in the food vacuole.

There have been a number of antimalarial discovery programs based on the benzimidazole core structure (Keri et al., 2015; L’Abbate et al., 2018; Attram et al., 2019; Devine et al., 2021). Screening of an in-house benzimidazole-based library revealed six pyrido-1,2-a-benzimidazole (PBI) hits with sub-100 nM potency against asexual blood stage (TCP-1) *P. falciparum* (Leshabane et al., 2021). Within these six hits, PBI.109 and PBI.105 showed nanomolar activity against early and late gametocytes (TCP-5) respectively, indicating they may have transmission-blocking potential (**Figure 1**). PBIs were found to accumulate in the food vacuole and inhibit the formation of hemozoin in asexual blood stage parasites (Singh et al., 2017; Korkor et al., 2020). However, haem detoxification is not critical for gametocyte development, suggesting an alternative MOA for their transmission-blocking activity (van Biljon et al., 2019). Another series of benzimidazole derivatives that were structurally related to PBI.109 and PBI.105 were found to inhibit microtubule formation in *P. falciparum* (Dziwornu et al., 2021). In *P. falciparum*, microtubules are crucial for cell division during asexual blood stage and critical for maintaining the inner membrane functionality for gametocyte development (Gerald et al., 2011; Parkyn Schneider et al., 2017). Thus, it is possible that the transmission-blocking activity of these PBI compounds stems from their ability to disrupt gametocyte microtubule function.

## Concluding remarks

Malaria eradication is a challenging mission, and resistance to all frontline antimalarials has made it more difficult. The COVID-19 pandemic has brought additional pressure to healthcare systems in malaria-endemic countries that often have pre-existing economic challenges. Significant efforts have been invested on a global level in screening, designing and repurposing medicines in order to find promising antimalarial candidates with multi-stage activity and novel MOAs. Here we have compiled a summary of recently reported antimalarial hit compounds with such properties (**Table 1**). Phenotypic screening has demonstrated the ability to identify antimalarial compounds with potent activity, and technological advances have expanded the screening of antimalarial activity to parasite lifecycle stages other than the symptomatic blood stage. However, there are still limitations in the current screening technologies and models. For a few antimalarial hits in this review, the liver-stage activities (TCP-4) against *P. falciparum* parasites were inferred from activity against liver-stage *P. berghei* parasites. Further development is needed to design more screening systems and models to improve the translation between *in vitro* pharmacology and clinical relevance. Drug discovery requires significant investment of time and resources, and there is no guarantee that the antimalarial hit compounds discussed in this review will reach clinical practice. However, target identification studies of these hit compounds have demonstrated promising results, yielding new drug targets and revealing novel biological pathways that are suitable for future drug discovery efforts. These mechanistic insights can underpin further chemical modification of these hits in order to optimize the activity, toxicity and pharmacokinetic profiles of these compounds with the goal to produce attractive antimalarial candidates for pre-clinical and clinical development.

## Author contributions

A-PL: Conceptualization, Formal analysis, Investigation, Visualization, Writing – original draft. CG: Writing – review & editing. GS: Supervision, Writing – review & editing. DC: Funding acquisition, Supervision, Writing – review & editing.
